# Personalized Therapy of Sarcomas

**DOI:** 10.3390/cancers15205110

**Published:** 2023-10-23

**Authors:** Ingrid M. E. Desar, Christian Rothermundt

**Affiliations:** 1 Department of Medical Oncology, Radboud University Medical Centre, 6500 HB Nijmegen, The Netherlands; 2 Department of Oncology and Hematology, Cantonal Hospital St. Gallen, 9007 St. Gallen, Switzerland; christian.rothermundt@kssg.ch

Sarcomas are a group of rare malignant mesenchymal tumors. Over 100 different histological subtypes already exist, and the number of subtypes continues to increase as advancements, particularly in molecular diagnostics, lead to further subtyping. Furthermore, sarcomas can occur at any anatomical site and at any age, and they can present with a wide range of symptoms, biological behaviors and prognoses. The heterogeneity of sarcomas is reflected in this Special Issue entitled “Personalized Therapy of Sarcomas”.

The systemic treatment options for gastrointestinal stromal tumors (GISTs) are reviewed by Golčić et al. In a disease that previously had a very poor prognosis due to its insensitivity to cytotoxic therapy, the outcome has improved dramatically following the discovery of driver mutations in cKIT, PDGFR and, to a lesser extent, BRAF, NF1 and SDH. The tyrosine kinase inhibitors (TKIs) imatinib, sunitinib, regorafenib, ripretinib and avapritinib are now available treatment options for GIST and are critically discussed in terms of their use in certain mutation types and their efficacy. The authors recommend the consideration of adverse events and quality of life in the decision-making process, as these TKIs are used over a long period of time, and the toxicity profiles of the drugs differ. In addition, complementary therapies such as surgery, radiotherapy and radiofrequency ablation should be discussed on a case-to-case basis among a multidisciplinary team.

Therapeutic drug monitoring (TDM) refers to the optimization of the individual dose of an oral TKI based on plasma levels in order to increase efficacy and avoid unnecessary toxicity. Flat-dose imatinib is investigated in all large clinical trials for locally advanced or metastatic GIST, while plasma levels are outside the therapeutic window in a large proportion of patients. However, there is a lack of prospective randomized data on fixed-dose versus TDM-guided dosing and on the significance of plasma levels versus intratumoral imatinib concentrations. This latter knowledge gap is addressed by Giraud et al. in an exploratory study. Their aim was to determine the correlation between plasma and tumor concentrations of imatinib in the neoadjuvant setting. In twenty-four GIST samples, they found an accumulation of imatinib in tumors without a clear distribution pattern. No correlation was found between plasma and tumor concentrations, nor with pathological response. While imatinib plasma concentrations are highly correlated with efficacy in the literature, this is not the case for intratumoral concentrations.

Another approach to better understand the efficacy of treatment in sarcomas is reported by van Ravensteijn et al. They investigated the prognostic value of magnetic resonance imaging (MRI) before and after neoadjuvant radiotherapy in 40 patients with myxofibrosarcoma. In particular, a tail sign, reflecting the typical infiltrative growth of myxofibrosarcomas, and a vascular pedicle were associated with worse disease-free survival. The increased percentage of necrosis on post radiotherapy MRIs was not associated with survival outcomes. Of note, they used MRIs from everyday clinical practice, as superior diffusion-weighted (DWI) MRI has not yet been implemented as standard of care. Data on the percentage of tumor necrosis in the pathologic specimen in STS relative to clinical outcomes are conflicting [1]. Further research on DWI and perfusion MRI to evaluate the efficacy of neoadjuvant radiotherapy would be interesting, including the ability of these techniques to identify patients at risk of early recurrence.

The heterogeneity of angiosarcomas was further investigated in a large cohort of primary (n = 79) and secondary (n = 178) angiosarcomas, illustrating that even within a sarcoma subtype, large diversity exists. Aberrations in the DNA damage repair (DDR) pathway combined with a T-cell-infiltrated micro-environment suggest a role for immunotherapy at least in secondary angiosarcomas.

The promising work of Tamiya et al. takes a step further away from the clinics. Ferroptosis is a new type of cell death mediated by ferrous iron. Ferroptosis is more sensitive in most of the sarcoma cell lines. The oncogenic factors transferrin receptor 1 (TFRC) and SHANK-associated RH domain interactor (SHARPIN) were highly expressed, especially in synovial sarcoma cell lines. SHARPIN was also shown to positively regulate ferroptosis through PGC1α reduction, mediated by nuclear factor-kappa B (NF-κΒ) and protein arginine methyltransferase 5 (PRMT5). Targeting ferroptosis may be a novel therapeutic approach in sarcoma and warrants further investigation.

The SURVSARC study concludes that more attention should be paid to fear of recurrence in personalized sarcoma survivorship. In over 1000 sarcoma patients, the prevalence of high fear of recurrence was as high as 45%. Patients with a high fear of recurrence also scored lower on the global health status scale. Telemedicine may help with these issues. After reviewing experiences with telemedicine beyond the COVID-19 period, Tsagkaris et al. report that the use of telemedicine in the management of sarcomas has led to improved clinical and psychological outcomes. Telehealth has been proven to be effective in a wide spectrum of applications, from consultations on physical therapy and psychological support to virtual care symptom management to the satisfaction of both patients and healthcare providers.

It is exciting to see the different approaches to personalizing sarcoma treatment that exist and are now published in this Special Issue of *Cancers*.

## List of Contributions

1. Golčić, M.; Jones, R.L.; Huang, P.; Napolitano, A. Evaluation of Systemic Treatment Options for Gastrointestinal Stromal Tumours. *Cancers* **2023**, *15,* 4081.
2. Giraud, E.L.; de Jong, L.A.W.; van den Hombergh, E.; Kaal, S.E.J.; van Erp, N.P.; Desar, I.M.E. Measuring Tumour Imatinib Concentrations in Gastrointestinal Stromal Tumours: Relevant or Redundant? *Cancers* **2023**, *15*, 2875.
3. van Ravensteijn, S.G.; Nederkoorn, M.J.; Wal, T.C.; Versleijen-Jonkers, Y.M.; Braam, P.M.; Flucke, U.E.; Bonenkamp, J.J.; Bonenkamp, J.J.; Bonenkamp, J.J.; de Wilt, J.H.W.; Desar, I.M.E.; et al. The Prognostic Relevance of MRI Characteristics in Myxofibrosarcoma Patients Treated with Neoadjuvant Radiotherapy. *Cancers* **2023**, *15*, 2843.
4. van Ravensteijn, S.G.; Versleijen-Jonkers, Y.M.H.; Hillebrandt-Roeffen, M.H.S.; Weidema, M.E.; Nederkoorn, M.J.L.; Bol, K.F.; Gorris, M.A.J.; Verrijp, K.; Kroeze, L.I.; de Bitter, T.J.J.; et al. Immunological and Genomic Analysis Reveals Clinically Relevant Distinctions between Angiosarcoma Subgroups. *Cancers* **2022**, *14*, 5938.
5. Tamiya, H.; Urushihara, N.; Shizuma, K.; Ogawa, H.; Nakai, S.; Wakamatsu, T.; Takenaka, S.; Takenaka, S. SHARPIN Enhances Ferroptosis in Synovial Sarcoma Cells via NF-κB- and PRMT5-Mediated PGC1α Reduction. *Cancers* **2023**, *15*, 3484.
6. Pellegrini, I.; Drabbe, C.; Grünhagen, D.J.; Van de Sande, M.A.J.; de Haan, J.J.; Keymeulen, K.; Bonenkamp, J.J.; Van der Graaf, W.T.A.; Husson, O. Fear of Cancer Recurrence in Sarcoma Survivors: Results from the SURVSARC Study. *Cancers* **2022**, *14*, 6099.
